# Clinical Decision Making in Inflammatory Bowel Disease Mimics: Practice Management from Inflammatory Bowel Disease LIVE

**DOI:** 10.1093/crocol/otae022

**Published:** 2024-04-11

**Authors:** Hannah W Fiske, Christopher Ward, Samir A Shah, Stefan D Holubar, Badr Al-Bawardy, Edward L Barnes, David Binion, Matthew Bohm, Myron Brand, Kofi Clarke, Benjamin L Cohen, Raymond K Cross, Jeffrey Dueker, Michael Engels, Francis A Farraye, Sean Fine, Erin Forster, Jill Gaidos, Philip Ginsburg, Alka Goyal, John Hanson, Hans Herfath, Tracy Hull, Colleen R Kelly, Mark Lazarev, L Campbell Levy, Joanna Melia, Jessica Philpott, Taha Qazi, Corey A Siegel, Andrew Watson, Steven D Wexner, Emmanuelle D Williams, Miguel Regueiro

**Affiliations:** Department of Internal Medicine, The Warren Alpert Medical School of Brown University, Providence, RI, USA; Division of Gastroenterology, Lahey Hospital and Medical Center, Burlington, MA, USA; Gastroenterology Associates Inc., The Warren Alpert Medical School of Brown University, Providence, RI, USA; Department of Colon and Rectal Surgery, Cleveland Clinic, Cleveland, OH, USA; Division of Gastroenterology, Yale School of Medicine, New Haven, CT, USA; Division of Gastroenterology, King Faisal Specialist Hospital and Research Centre, Riyadh, Saudi Arabia; Division of Gastroenterology and Hepatology, University of North Carolina School of Medicine, Chapel Hill, NC, USA; Division of Gastroenterology, University of Pittsburgh Medical Center, Pittsburgh, PA, USA; Division of Gastroenterology, Indiana University School of Medicine, Carmel, IN, USA; Division of Gastroenterology, Yale School of Medicine, New Haven, CT, USA; Division of Gastroenterology, Penn State Health Milton S Hershey Medical Center, Hershey, PA, USA; Department of Gastroenterology, Hepatology, & Nutrition, Cleveland Clinic, Cleveland, OH, USA; Division of Gastroenterology & Hepatology, University of Maryland School of Medicine, Baltimore, MD, USA; Division of Gastroenterology, University of Pittsburgh Medical Center, Pittsburgh, PA, USA; Division of Gastroenterology, Lehigh Valley Health Network, Allentown, PA, USA; Division of Gastroenterology and Hepatology, Mayo Clinic, Jacksonville, FL, USA; Division of Gastroenterology, The Warren Alpert Medical School of Brown University, Providence, RI, USA; Division of Gastroenterology, Medical University of South Carolina, Charleston, SC, USA; Division of Gastroenterology, Yale School of Medicine, New Haven, CT, USA; Division of Gastroenterology, Yale School of Medicine, New Haven, CT, USA; Department of Pediatric Gastroenterology, Stanford University School of Medicine, Stanford, CA, USA; Division of Gastroenterology and Hepatology, Atrium Health, Charlotte, NC, USA; Division of Gastroenterology and Hepatology, University of North Carolina School of Medicine, Chapel Hill, NC, USA; Department of Colon and Rectal Surgery, Cleveland Clinic, Cleveland, OH, USA; Division of Gastroenterology, Brigham and Women’s Hospital, Boston, MA, USA; Division of Gastroenterology, Johns Hopkins Medical Center, Baltimore, MD, USA; Center for Digestive Health, Section of Gastroenterology and Hepatology, Dartmouth Hitchcock Medical Center, Lebanon, NH, USA; Division of Gastroenterology, Johns Hopkins Medical Center, Baltimore, MD, USA; Department of Gastroenterology, Hepatology, & Nutrition, Cleveland Clinic, Cleveland, OH, USA; Department of Gastroenterology, Hepatology, & Nutrition, Cleveland Clinic, Cleveland, OH, USA; Center for Digestive Health, Section of Gastroenterology and Hepatology, Dartmouth Hitchcock Medical Center, Lebanon, NH, USA; Department of Surgery, University of Pittsburgh Medical Center, Pittsburgh, PA, USA; Department of Colorectal Surgery, Cleveland Clinic Florida, Weston, FL, USA; Division of Gastroenterology, Penn State Health Milton S Hershey Medical Center, Hershey, PA, USA; Department of Gastroenterology, Hepatology, & Nutrition, Cleveland Clinic, Cleveland, OH, USA

**Keywords:** clinical decision-making, Crohn’s disease, inflammatory bowel disease (IBD), IBD mimics (IBD-M), ulcerative colitis

## Abstract

**Background:**

Since 2009, inflammatory bowel disease (IBD) specialists have utilized “IBD LIVE,” a weekly live video conference with a global audience, to discuss the multidisciplinary management of their most challenging cases. While most cases presented were confirmed IBD, a substantial number were diseases that mimic IBD. We have categorized all IBD LIVE cases and identified “IBD-mimics” with consequent clinical management implications.

**Methods:**

Cases have been recorded/archived since May 2018; we reviewed all 371 cases from May 2018–February 2023. IBD-mimics were analyzed/categorized according to their diagnostic and therapeutic workup.

**Results:**

Confirmed IBD cases made up 82.5% (306/371; 193 Crohn’s disease, 107 ulcerative colitis, and 6 IBD-unclassified). Sixty-five (17.5%) cases were found to be mimics, most commonly medication-induced (*n* = 8) or vasculitis (*n* = 7). The evaluations that ultimately resulted in correct diagnosis included additional endoscopic biopsies (*n* = 13, 21%), surgical exploration/pathology (*n* = 10, 16.5%), biopsies from outside the GI tract (*n* = 10, 16.5%), genetic/laboratory testing (*n* = 8, 13%), extensive review of patient history (*n* = 8, 13%), imaging (*n* = 5, 8%), balloon enteroscopy (*n* = 5, 8%), and capsule endoscopy (*n* = 2, 3%). Twenty-five patients (25/65, 38%) were treated with biologics for presumed IBD, 5 of whom subsequently experienced adverse events requiring discontinuation of the biologic. Many patients were prescribed steroids, azathioprine, mercaptopurine, or methotrexate, and 3 were trialed on tofacitinib.

**Conclusions:**

The diverse presentation of IBD and IBD-mimics necessitates periodic consideration of the differential diagnosis, and reassessment of treatment in presumed IBD patients without appropriate clinical response. The substantial differences and often conflicting treatment approaches to IBD versus IBD-mimics directly impact the quality and cost of patient care.

## Graphical Abstract

**Graphical Abstract:**
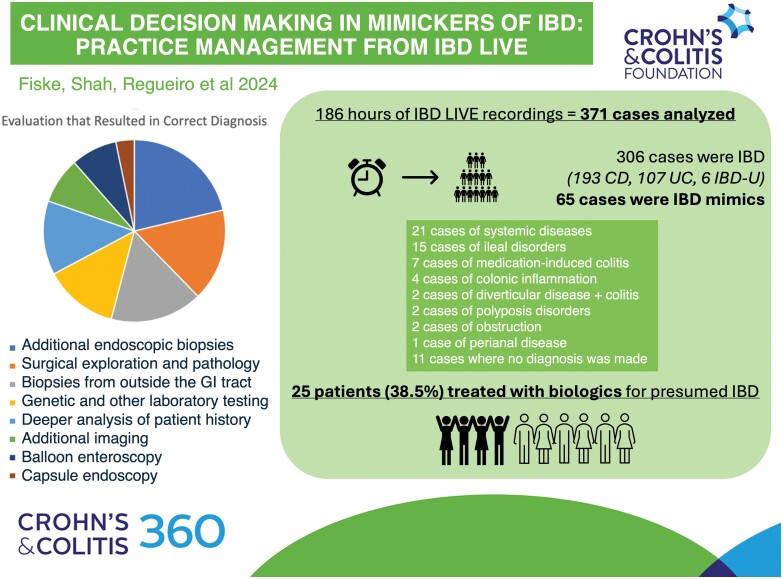

## Introduction

Inflammatory bowel disease (IBD) is an umbrella term used to define chronic gastrointestinal (GI) inflammation in both Crohn’s disease (CD) and ulcerative colitis (UC). The worldwide incidence and prevalence of both diseases have been increasing, and it is estimated that approximately 3 million Americans are currently diagnosed with IBD.^1–5^ Although similar in many ways, CD and UC have some key differences that distinguish them. UC is characterized by continuous mucosal inflammation that is limited to the colorectum, while CD occurs anywhere from the mouth to the anus, and is characterized by transmural inflammation with skip lesions, granulomas, and fistulae.^5–7^ Between 5% and 15% of IBD cases do not meet the strict criteria required for either CD or UC; these patients are cataloged as IBD-unclassified (IBD-U).^1,8,9^ Given the nonspecific symptomatology of IBD, there are numerous diseases that mimic CD or UC; these IBD-mimics (IBD-M) should be heavily considered on the differential when working up suspected IBD.

Currently, there is no gold standard test for the diagnosis of IBD. The diagnosis of IBD is based on clinical, endoscopic, histologic, radiologic, and biochemical investigations. However, establishing a diagnosis can be challenging in the setting of nonspecific symptomatology or variability in endoscopic or histologic findings. Adding to the complexity of diagnosis, up to 14% of patients classified as either CD or UC end up with a change in their diagnosis as their disease course progresses, most commonly from UC to CD following ileal pouch-anal anastomosis.^9^ Additionally, it has been estimated that 80% of patients with IBD-U will be reclassified within 8 years of diagnosis to either CD or UC.^10,11^ IBD-U patients are known to have a worse prognosis, likely because their lack of definitive diagnosis results in substandard management that is unable to be tailored to the specific disease at hand.^9,12^ As a further detriment to those given an IBD-U classification, patients without a clear diagnosis are typically excluded from therapeutic clinical trials.^9^

The approach to the diagnosis of IBD is complex, and there is a broad differential diagnosis. Patients’ symptoms may be continuous or intermittent and depend on the severity and extent of their disease. Co-infection with enteric pathogens can alter the presentation and obscure the clinical picture. For example, *Clostridioides difficile* infection (CDI) can mimic an IBD flare. Complicating the picture, patients with known IBD are at an increased risk of developing CDI, and simultaneously, patients with concomitant CDI are at a higher risk of IBD flares.^1^ It is crucial to pay close attention to the patient’s history, as it may point towards the ultimate diagnosis. Medications (most commonly non-steroidal anti-inflammatory drugs and immune-based chemotherapies) are often the source of gastrointestinal symptoms; patients with a history of malignancy are at heightened risk of radiation-induced colitis. Travel, family, and sexual histories are frequent clues to the correct diagnosis. Determining the diagnosis is important, as early appropriate therapy for IBD decreases complications and improves quality of life; however, if IBD is not the underlying etiology of the patient’s symptoms, the use of advanced IBD therapies may not result in symptomatic improvement and could exacerbate symptoms due to immune suppression.

Since 2009, IBD specialists have participated in a weekly live video conference called “IBD LIVE,” where they discuss the multidisciplinary management of their most interesting and challenging cases.^13–22^ This conference features robust interactive case discussions between gastroenterologists and colorectal surgeons from multiple academic institutions and private practices, and hosts a global audience of 150–200 each week. The objective of most case presentations is to elicit recommendations for further evaluation or the next steps in treatment; however, at times, the question is regarding the underlying diagnosis. We retrospectively categorized all IBD LIVE cases and identified cases of IBD-M with consequent clinical management implications. Herein, we explore the prevalence of various IBD-M and describe the evaluation that ultimately led to the diagnosis.

## Materials and Methods

We performed a quantitative review of IBD-M presented at IBD LIVE. Although IBD LIVE originated in 2009, cases were not recorded, transcribed, or archived until May 2018.^13,20^ From May 2018 through February 2023, the transcripts from a total of 186 hours of case conferences featuring 371 discussed cases were reviewed and cataloged. Cases of IBD-M were defined as those with features of IBD that ultimately resulted in a non-IBD diagnosis. Specifics were thoroughly documented for each IBD-M case, including the original suspected diagnosis, diagnostic workup conducted, various treatments attempted, and evaluation that led to the correct diagnosis. The cases were categorized based on ultimate diagnosis.

Our primary objective was to assess the ultimate diagnosis and presence of IBD-M. Further analysis was carried out only for those patients determined to have a true IBD-M, or for those with a known diagnosis of IBD who developed a mimic on top of their disease, masquerading as an IBD flare. Secondary outcomes included diagnostic workup, examination of the often invasive and extensive testing that patients underwent, and treatments attempted, exploring those who were exposed to biologics or small molecules.

## Ethical Considerations

This study is original and has not been published previously. All authors significantly contributed to the design of the study, data analysis, drafting, and approved the final version of the manuscript. All authors agree to be accountable for all aspects of this study.

## Results

Over a 5-year period, 306 of the 371 cases discussed (82.5%) had an IBD diagnosis including 193/306 (63%) CD cases, 107/306 (35%) UC cases, and 6/306 (2%) IBD-U. The remaining 65 (17.5%) cases were IBD-M, referred to an IBD specialist with a presumed diagnosis of IBD (Table 1). These 65 cases of IBD-M were presented by IBD specialists from 19 different hospitals, practices, or academic centers—18 (94.7%) within the United States and 1 international.

**Table 1.** IBD Mimics presented at IBD LIVE, 2018–2023

Category	Mimic	*n* =	Diagnoses
Systemic diseases	CD	21	Vasculitis (7), CVID (2), Sweets (1), EGPA (1), CHAI (1), BADAS (1), Behcet’s (1), eosinophilic esophagitis (1), carcinoid (1), histoplasmosis (1), sarcoidosis (1), amyloidosis (1), PIK3cd (1), adrenal insufficiency (1)
Ileal disorders	CD	15	BCL (4), IMHMV (2), follicular lymphoma (1), T-cell lymphoma (1), Kaposi’s sarcoma (1), Meckel’s diverticulum (1), pouchitis (1), appendiceal carcinoid (1), tropical sprue (1), collagenous sprue (1), and autoimmune enteropathy (1)
Medication-induced	UC/CD	7	**UC:** Immune checkpoint inhibitor (Pembrolizumab [1], Ipilimumab + Nivolumab [2]) **CD:** Chemotherapy (Encorafenib + Binimetinib [1]), Olmesartan (1), Mycophenolate (1), mu-opioid receptor agonist (Kratom) + GABA-mimetic (Phenibut; 1)
Colonic inflammation	UC	4	Ischemic colitis (1), diversion colitis (1), CMV colitis (1), STI proctitis (1)
Diverticular disease + colitis	CD	2	SCAD (2)
Polyposis disorders	UC	2	Cronkhite-Canada syndrome (1), juvenile polyposis syndrome (1)
Obstruction	CD	2	Abdominal actinomyces (1), cecal volvulus (1)
Perianal disease	CD	1	Cryptoglandular abscess (1)
No diagnosis made	UC/CD	11	Unclear at time of IBD LIVE (9), presumed C-MUSE (1), presumed congenital tailgut cyst (1)

BADAS, bowel-associated dermatosis-arthritis syndrome; BCL, B-cell lymphoma; CD, Crohn’s disease; CHAI, CTLA-4 haploinsufficiency with autoimmune infiltrates; C-MUSE, cryptogenic multifocal ulcerous stenosing enteritis; CMV, cytomegalovirus; CVID, common variable immunodeficiency; EGPA, eosinophilic granulomatosis with polyangiitis; GABA, gamma-aminobutyric acid; IBD, inflammatory bowel disease; IMHMV, idiopathic myointimal hyperplasia of the mesenteric veins; PIK3cd, phosphatidylinositol-4,5-bisphosphonate 3-kinase catalytic subunit delta; SCAD, segmental colitis associated with diverticulosis; STI, sexually transmitted infection; UC, ulcerative colitis.

### Crohn’s Mimics

There were 21/65 (32.3%) cases of systemic diseases misdiagnosed as CD, including vasculitis (*n* = 7) and common variable immune deficiency ( *n* = 2), among numerous others (Table 1).

There were 15/65 (23.1%) cases of ileal disorders misdiagnosed as CD, which were ultimately diagnosed as B-cell lymphoma (*n* = 4), idiopathic myointimal hyperplasia of mesenteric veins (*n* = 2), and several others (Table 1).

There were 4/65 (6.2%) cases of medication-induced disease, originally diagnosed as CD, ultimately determined to be related to chemotherapy, olmesartan, mycophenolate, and a combination of kratom and the GABA-mimetic phenibut.

There were 2/65 (3.1%) patients with diverticular disease and colonic inflammation, originally thought to be CD, but determined to be segmental colitis associated with diverticulitis (SCAD).

There were 2/65 (3.1%) patients with obstruction, originally thought to have CD, but who were ultimately diagnosed with abdominal actinomyces and cecal volvulus.

There was 1/65 (1.7%) case of perianal disease, misdiagnosed as CD, and later diagnosed as cryptoglandular abscess.

### UC Mimics

There were 4/65 (6.2%) cases of colonic inflammation that were misdiagnosed as UC, including ischemic colitis, diversion colitis, cytomegalovirus (CMV) colitis, and sexually transmitted proctitis.

There were 3/65 (4.6%) cases of medication-induced disease, originally diagnosed as UC, and ultimately determined to be related to the immune checkpoint inhibitors ipilimumab and nivolumab (*n* = 2) and pembrolizumab (*n* = 1).

There were 2/65 (3.1%) cases of polyposis disorders misdiagnosed as UC, including one patient with Cronkhite-Canada syndrome and one patient with juvenile polyposis syndrome.

### Other

Finally, 11/65 (16.9%) cases had no consensus diagnosis, with broad differential diagnoses including cryptogenic multifocal ulcerous stenosing enteritis (C-MUSE) and a presumed congenital tailgut cyst.

### Diagnostic Workup

For each IBD-M case, further evaluation was completed with imaging, additional endoscopy, and other diagnostic modalities described below. Numbers and percentages that follow here refer to the workup obtained during the acute presentation and did not include any diagnostic workup obtained prior to the presentation of the mimic.

Imaging included computed tomography (CT) scan (*n* = 58/65, 89%), CT enterography (n = 10, 15%), CT angiography (*n* = 7, 11%), positron emission tomography scan (*n* = 6, 9%), magnetic resonance imaging (*n* = 28, 43%), magnetic resonance enterography (*n* = 22, 34%), magnetic resonance angiography (*n* = 1, 1.5%), magnetic resonance cholangiopancreatography (n = 1, 1.5%), video capsule endoscopy (*n* = 14, 22%), timed barium esophagram (*n* = 3, 5%), small bowel follow through (*n* = 3, 5%), abdominal ultrasound (*n* = 4, 6%), abdominal x-ray (*n* = 3, 5%), hepatobiliary iminodiacetic acid scan (*n* = 1, 1.5%), and interventional radiography angiography (*n* = 1, 1.5%).

The procedures performed included colonoscopy (*n* = 62, 95%), upper endoscopy (EGD; *n* = 37, 57%), small bowel enteroscopy (via spiral, single balloon, or double balloon; *n* = 13, 20%), flexible sigmoidoscopy (*n* = 12, 18%), laparoscopic procedures (*n* = 15, 23%), endoscopic ultrasound (EUS; n = 4, 6%), pouchoscopy (*n* = 2, 3%), and exam under anesthesia (*n* = 2, 3%). Biopsies were taken from the colon, esophagus, small bowel, liver, lymph nodes, parotid gland, and skin.

**Figure 1.**
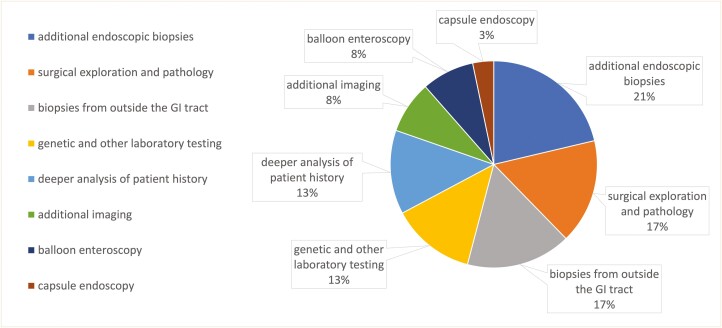
Evaluation that resulted in correct diagnosis of inflammatory bowel disease (IBD) Mimics presented at IBD LIVE, 2018–2023.

The evaluations that ultimately resulted in the correct diagnosis included (Figure 1): additional endoscopic biopsies (*n* = 13, 21%), surgical exploration and pathology (*n* = 10, 16.5%), biopsies from outside the GI tract (*n* = 10, 16.5%), genetic and other laboratory testing (*n* = 8, 13%), deeper analysis of patient history, including medication review, family, social, and sexual history (*n* = 8, 13%), additional imaging (*n* = 5, 8%), balloon enteroscopy (*n* = 5, 8%), and capsule endoscopy (*n* = 2, 3%).

Of the 65 cases of IBD-M discussed at IBD LIVE, 25 remained undiagnosed at the time of presentation at the conference. Of those, 14/25 (56%) successfully received a diagnosis of a specific IBD-M following additional workup which was prompted by the collaborative discussions during IBD LIVE. The remaining 11/25 (44%) still lack a clear diagnosis, as discussed above.

### Treatment

**Figure 2.**
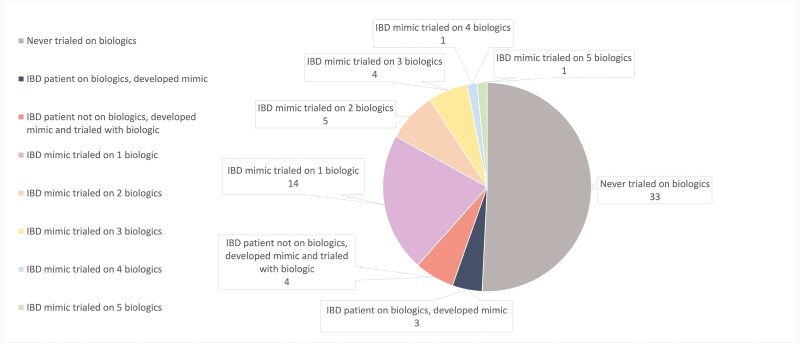
Biologic Usage in inflammatory bowel disease (IBD) Mimics presented at IBD LIVE, 2018–2023.

**Figure 3.**
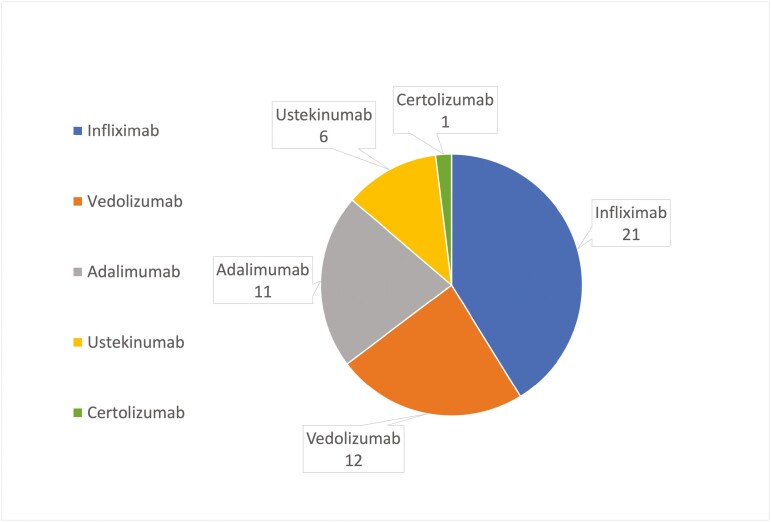
Biologics Used for inflammatory bowel disease (IBD) Mimics presented at IBD LIVE, 2018–2023.

Prior to being diagnosed as a case of IBD-M, many patients were prescribed advanced therapies for the treatment of their presumed diagnosis of IBD. Several of the IBD-M patients were prescribed steroids, azathioprine, mercaptopurine, or methotrexate, and 3 were treated with tofacitinib (for Sweet’s syndrome, PIK3cd, and SCAD); 25 of the 65 mimics (38.5%) were treated with biologics for presumed IBD (Table 2, Figure 2); 14 were treated with one biologic, 5 with 2 biologics, and 6 with 3 or more biologics. The biologics used in the management of these IBD-M (Figure 3) included infliximab (*n* = 21), vedolizumab (*n* = 12), adalimumab (*n* = 11), ustekinumab (*n* = 6), and certolizumab (*n* = 1).

**Table 2.** Biologics used for IBD Mimics presented at IBD LIVE, 2018–2023

Category	*n* =	Diagnoses
Never trialed on biologics	33	*Varied IBD mimics (* Table 1 *)*
IBD patient in remission on biologics, then developed mimic	3	Cecal volvulus, disseminated histoplasmosis, and adrenal insufficiency
IBD patient in remission NOT on biologics, then developed mimic which was treated with biologics	4	Medication-induced, eosinophilic esophagitis, immune-mediated esophagitis, and ischemic colitis
IBD mimic trialed on biologics	25	
1 biologic	14	Medication-induced (2), amyloidosis, sarcoidosis, Behcet’s, Sweet syndrome, vasculitis, B-cell lymphoma, T-cell lymphoma, Kaposi’s sarcoma, IMHMV, SCAD, diversion colitis, and autoimmune enteropathy
2 biologics	5	Medication-induced (2), angioedema, infectious, and bacterial overgrowth
3 biologics	4	SCAD, PNH, ischemic pouchitis, and juvenile polyposis
4 biologics	1	PIK3cd
5 biologics	1	CHAI

CHAI, CTLA-4 haploinsufficiency with autoimmune infiltrates; IBD, inflammatory bowel disease; IMHMV, idiopathic myointimal hyperplasia of the mesenteric veins; PIK3cd, phosphatidylinositol-4,5-bisphosphonate 3-kinase catalytic subunit delta; PNH, paroxysmal nocturnal hemoglobinuria; SCAD, segmental colitis associated with diverticulosis.

Of the 25 (38.5%) cases of biologic usage without definitive confirmation of IBD, 6/25 (24%) were ultimately diagnosed with a condition for which biologics were an appropriate treatment. These mimics included drug-induced colitis (*n* = 3), Behcet’s disease (*n* = 1), SCAD (*n* = 1), and CHAI (*n* = 1). There were 3 patients with confirmed IBD, in remission on a biologic, who subsequently had symptoms concerning an IBD flare. In each of these cases, IBD was not the cause of their symptoms (symptoms determined to be due to cecal volvulus, disseminated histoplasmosis, and adrenal insufficiency). There were 4 patients with confirmed IBD, in remission off biologics, who later developed symptoms suspicious for an IBD flare. Each of these patients was started on biologics to treat the supposed flare; however, each case was ultimately found to be an IBD-M (symptoms determined to be due to eosinophilic esophagitis, ischemic colitis, drug-induced colitis, and immune-mediated esophagitis). Five of the twenty-five patients who were started on biologics (25%) subsequently experienced adverse events requiring discontinuation of biologics. A total of 33/65 (50.8%) IBD-M were never treated with biologics.

## Discussion

In this study, we analyzed 5 years of patient cases presented at IBD LIVE, and identified that 17.5% of cases (65/371) were IBD-M. Of these, 45 (69.2%) were CD mimics, 9 (13.8%) were UC mimics, and 11 (16.9%) remained undiagnosed at the time of this study. In many cases, the final diagnosis was reached via additional endoscopic biopsies, surgical exploration with pathology, or biopsies from sites external to the GI tract (Figure 1). The most common mimics included medication-induced diseases, vasculitis, and lymphoma (Table 1). Many IBD-M patients originally received advanced therapies, including 38.5% (25/65) who were treated with empiric biologics for presumed CD or UC. In the majority of these cases, biologics were given in the setting of a working diagnosis of difficult-to-manage IBD, prompting the provider to consider a therapeutic trial with a biologic. Many of the patients had been put on biologics by their local gastroenterologist prior to being referred to the IBD center with a presumed diagnosis of IBD; it was not until after additional workup was completed that the true diagnosis of IBD-M was revealed, and the biologic was stopped.

The diverse presentation of signs and symptoms of IBD and the complexity of both assessment and diagnosis necessitate the significant consideration of IBD-M. Patients may be misdiagnosed with IBD for many years before being correctly diagnosed and managed, resulting in inappropriate treatment of their disease. Clinicians should consider these mimics due to their significant implications on quality of life and harm reduction, with the potential to avoid the use of biological therapies in the management of IBD. The substantial differences and often conflicting treatment approaches to IBD versus IBD-M directly impact the quality and cost of patient care. This study highlights the importance of reassessment of advanced therapy in patients without a response. Often treatment needs to be optimized or changed for IBD, but in some cases, the diagnosis of IBD should questioned.

We determined 3 primary categories of diagnostic testing that ultimately yielded the correct diagnosis: (1) procurement of additional tissue for evaluation, (2) different radiographic or endoscopic evaluation from the original workup, especially inspection of the small bowel, and (3) more thorough history-taking.

Our study had several limitations. We recognize that the IBD LIVE cases represent a skewed sample that does not directly represent the worldwide population of IBD patients or the population of IBD patients typically seen in community practice, as each presented case was chosen for its challenging or unique nature. Hence there is selection bias both in that only the most challenging cases were chosen to be presented, and in that the cases were selected from centers and private practices with significant IBD volume and expertise. Additionally, mimics may be more likely to be presented in order to try to “stump” colleagues or share how the diagnosis was ultimately made. While there is no clear consensus on the true incidence of IBD-M in the population, our study should not be considered a representative population sample; the 17.5% rate of IBD-M in our study is likely significantly higher than that of the general population of IBD patients. We would also like to highlight that IBD LIVE is a meeting of gastroenterologists from a series of tertiary care referral centers with significant clinical, radiographic, and pathologic IBD expertise; we acknowledge that the resources available to these physicians are not always readily available to the general GI practitioner. However, given that the cases presented at IBD LIVE were often patients who were referred to the IBD center by their local community GI doctor, we believe that this list is a representative sample of some of the more challenging and difficult-to-diagnose cases that may be encountered by the general gastroenterologist. One additional limitation is that the case discussion format does not include a detailed analysis of the pathologic data, although all the sites had access to GI-trained pathologists. There are several studies that have identified IBD-M according to histopathology^.11,23–26^

## Conclusion

In conclusion, there are a number of inflammatory GI conditions that mimic CD or UC. In patients with presumptive IBD who do not respond to therapy and have a change in their disease course, clinicians should consider other differential diagnostic possibilities before proceeding to advanced therapies. The list of IBD-Ms generated from IBD LIVE (Table 1) can help clinicians broaden their differential diagnosis when seeing a patient with presumed IBD. Diagnosing IBD can be challenging due to nonspecific symptomatology and variability in endoscopic and histologic findings, which can overlap with numerous IBD-M. We hope that sharing our experience with IBD-M will help others consider these entities and arrive at the correct diagnosis to guide appropriate therapy.

## Data Availability

The data generated during this study are available from the corresponding author upon reasonable request.
